# Narrative review of methods and findings of recent studies on the carriage of meningococci and other *Neisseria* species in the African Meningitis Belt

**DOI:** 10.1111/tmi.13185

**Published:** 2018-12-06

**Authors:** Brian M. Greenwood, Abraham Aseffa, Dominique A. Caugant, Kanny Diallo, Paul A. Kristiansen, Martin C. J. Maiden, James M. Stuart, Caroline L. Trotter

**Affiliations:** ^1^ London School of Hygiene & Tropical Medicine London UK; ^2^ Armauer Hansen Research Institute Addis Ababa Ethiopia; ^3^ Norwegian Institute of Public Health Oslo Norway; ^4^ Department of Bacteriology Noguchi Memorial Research Institute University of Legon Accra Legon; ^5^ Department of Zoology University of Oxford Oxford UK; ^6^ Department of Veterinary Medicine University of Cambridge Cambridge UK

**Keywords:** *Neisseria meningitidis*, meningococcal carriage, African meningitis belt, *Neisseria meningitidis*, portage du méningocoque, ceinture africaine de la méningite

## Abstract

**Objective:**

To review the findings of studies of pharyngeal carriage of *Neisseria meningitidis* and related species conducted in the African meningitis belt since a previous review published in 2007.

**Methods:**

PubMed and Web of Science were searched in July 2018 using the terms ‘meningococcal OR *Neisseria meningitidis* OR *lactamica* AND carriage AND Africa’, with the search limited to papers published on or after 1st January 2007. We conducted a narrative review of these publications.

**Results:**

One hundred and thirteen papers were identified using the search terms described above, 20 of which reported new data from surveys conducted in an African meningitis belt country. These papers described 40 surveys conducted before the introduction of the group A meningococcal conjugate vaccine (MenAfriVac^R^) during which 66 707 pharyngeal swabs were obtained. Carriage prevalence of *N. meningitidis* varied substantially by time and place, ranging from <1% to 24%. The mean pharyngeal carriage prevalence of *N. meningitidis* across all surveys was 4.5% [95% CI: 3.4%, 6.8%] and that of capsulated *N. meningitidis* was 2.8% [95% CI: 1.9%; 5.2%]. A study of households provided strong evidence for meningococcal transmission within and outside households. The introduction of MenAfriVac^®^ led to marked reductions in carriage of the serogroup A meningococcus in Burkina Faso and Chad.

**Conclusions:**

Recent studies employing standardised methods confirm the findings of older studies that carriage of *N. meningitidis* in the African meningitis belt is highly variable over time and place, but generally occurs with a lower prevalence and shorter duration than reported from industrialised countries.

## Abstract (fr)

**Objectif:**

Passer en revue les résultats d’études sur le portage pharyngé de *Neisseria meningitidis* et des espèces apparentées, menées dans la ceinture africaine de la méningite depuis un précédent rapport publié en 2007.

**Méthodes:**

Des recherches sur PubMed et Web of Science ont été effectuées en juillet 2018 en utilisant les termes «méningocoque OU *Neisseria meningitidis* OU *lactamica* ET portage ET Afrique». La recherche a été limitée aux articles publiés à partir du 1(^er^ janvier 2007. Nous avons effectué une analyse narrative de ces publications.

**Résultats:**

113 articles ont été identifiés en utilisant les termes de recherche décrits ci‐dessus, dont 20 rapportaient de nouvelles données provenant d'enquêtes menées dans un pays africain de la ceinture de la méningite. Ces articles décrivaient 40 enquêtes menées avant l'introduction du vaccin conjugué contre le méningocoque du groupe A (MenAfriVac®), au cours de laquelle 66.707 prélèvements pharyngés sur écouvillons ont été obtenus. La prévalence de portage de *N. meningitidis* variait considérablement selon le temps et le lieu, allant de <1% à 24%. La prévalence moyenne de portage pharyngé de *N. meningitidis* dans toutes les enquêtes était de 4,5% [IC95%: 3,4% à 6,8%] et de 2,8% pour celle de *N. meningitidis* encapsulée [IC95%: 1,9% à 5,2%]. Une étude sur les ménages a fourni des preuves solides de la transmission du méningocoque à l'intérieur et à l'extérieur des ménages. L'introduction de MenAfriVac® a entraîné une réduction marquée du portage du méningocoque du sérogroupe A au Burkina Faso et au Tchad.

**Conclusions:**

Des études récentes utilisant des méthodes standardisées confirment les résultats d’études antérieures selon lesquels le portage de *N. meningitidis* dans la ceinture africaine de la méningite est très variable dans le temps et le lieu, mais survient généralement avec une prévalence inférieure et une durée plus courte que celle rapportée dans les pays industrialisés.

## Introduction

Only a very small proportion of infections caused by *Neisseria meningitidis* progress to invasive meningococcal disease, the majority resulting in an asymptomatic, or only mildly symptomatic, infection of the pharynx. Study of pharyngeal meningococcal carriage is, therefore, essential for a full understanding of the epidemiology of this infection 1. Furthermore, there is now good evidence that the impact of serogroups A and C meningococcal conjugate vaccines is due in a large part to their ability to prevent carriage 2, 3, 4, 5, 6. Therefore, there has been a renewed interest in pharyngeal carriage of *Neisseria* species in the African meningitis belt where the greatest burden of meningococcal disease still occurs.

A review of studies of meningococcal carriage conducted in the African meningitis belt between 1915 and 2007 was published in 2007 7. This review noted large variations in meningococcal carriage prevalence and serogroup between places and over time. How much of this variation was due to differences in the field techniques used to collect specimens and/or in the laboratory methods used to analyse these samples, and how much was a reflection of true variation in the epidemiology of this infection, were uncertain. Since 2007, additional studies of meningococcal carriage have been undertaken in countries of the African meningitis belt using similar, more standardised, field and laboratory methods. A narrative review of these studies and the ways in which their findings contribute to our understanding of the overall epidemiology of *N*. *meningitidis* are presented here.

## Methods

**Figure 1:**
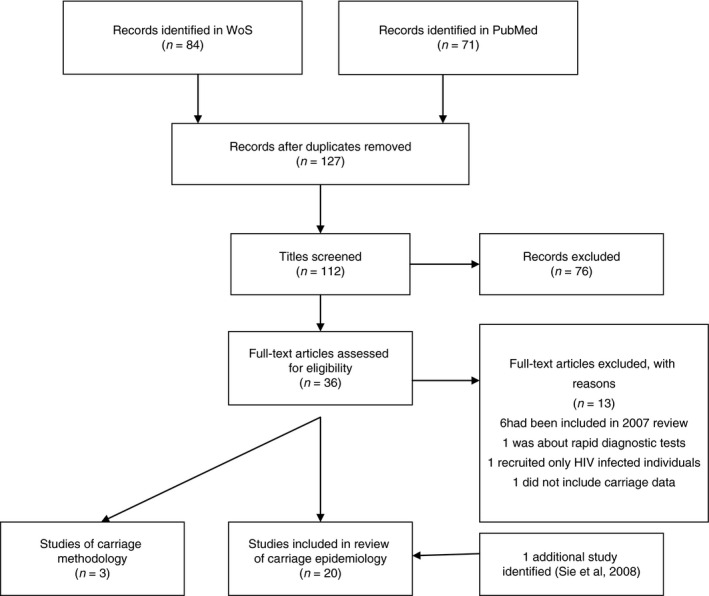
Search methods employed to identify papers published since 2007 relevant to carriage of meningococci in the African meningitis belt.

We searched PubMed and Web of Science on 4th July 2018 using the terms ‘meningococcal OR *Neisseria meningitidis* OR *lactamica* AND carriage AND Africa’, restricting our search to papers published on or after 1st January 2007 (Figure 1). This search identified 113 papers, 20 of which reported new data on meningococcal carriage in representative populations in countries of the African meningitis belt (Table S1). No further relevant documents from the grey literature were found on the System for Information on Grey Literature in Europe (SIGLE).

## Results

Twenty papers were identified which described meningococcal carriage surveys conducted in nine African meningitis belt countries and reported since 2007 4, 5, 6, 8, 9, 10, 11, 12, 13, 14, 15, 16, 17, 18, 19, 20, 21, 22, 23, 24. Sixteen of these surveys were conducted prior to the deployment of the group A meningococcal conjugate vaccine (MenAfriVac^®^). Three additional papers reported specifically on methods used in the MenAfriCar 25, 26 and Burkina Faso 27 studies. The methods used in these studies and their findings are summarised in this paper.

### Methods

#### Selection of study participants

Participants were selected for the 20 surveys conducted by the MenAfriCar Consortium randomly from within a population defined either through an established Demographic Surveillance System (DSS) or from a population census 25. A random sample of subjects in each of four age categories (0–4, 5–14, 15–29 and ≥30 years) was chosen within selected households. In Burkina Faso, surveys were conducted in both rural and urban areas. In the rural districts, 10 villages were selected based on probability proportional to size, and within each village households were chosen randomly using Global Positioning System coordinates. In the urban district, city blocks were randomly chosen based on existing maps and all subjects living in selected households were invited to participate. In Ethiopia, a DSS was used to select a random sample of subjects in a region different from that studied by the MenAfriCar Consortium 18. In the remainder of the studies, a convenience sample was used.

The majority of surveys were restricted to subjects aged 1–29; only the studies conducted by the MenAfriCar Consortium included subjects in all age groups including infants.

#### Swabbing technique

Since the time of the 2007 review 7, standardisation of swabbing techniques has improved with nearly all surveys undertaking swabbing of the back of the throat using a swab introduced through the mouth. This approach was used in all the studies reviewed except for one survey 23, which used swabbing of the posterior nasopharynx with a swab introduced through the nose, the technique used most frequently for surveys of pneumococcal carriage. Among studies that used the oropharyngeal approach, swabbing of solely the posterior pharyngeal wall was used in two studies whilst in the remainder both the posterior pharynx and the tonsils were swabbed. A comparative study conducted in Mali showed no significant difference in the prevalence of pharyngeal carriage of *N. meningitidis* using either of these two methods 26.

#### Identification of *Neisseria meningitidis*

Swabs were inoculated directly onto selective agar plates in all but one study, which employed transport medium 23, and plates were incubated within a maximum of 6 h after swabbing. The most widely used selective medium was Modified Thayer Martin containing vancomycin, colistin, nystatin and trimethoprim. Plates were incubated for 24–48 h at 37 °C in a 5% CO_2_ atmosphere.

All studies relied initially on conventional microbiological approaches to identify *Neisseria* species. An alternative method of identifying meningococcal carriage was evaluated in The Gambia; culture of the swab in Todd‐Hewitt broth followed by DNA extraction and quantitative real‐time PCR. This method had a higher sensitivity than conventional microbiological methods 20.

Serogrouping was performed initially by slide agglutination. Six of the seven countries involved in the MenAfriCar studies used PCR to confirm the identity of *N. meningitidis* isolates and to identify the genes coding for the capsule 25. Confirmatory analyses and molecular characterisation were performed in external laboratories in Europe. Most studies also reported the variants of the two outer membrane proteins PorA and FetA. Studies in Burkina Faso and Ethiopia reported sequence types 14, 22 and the most recent studies also used whole genome sequencing (WGS) 21, 22.

A laboratory quality control system was established for the later carriage studies conducted in Burkina Faso 27 and a similar quality control scheme was employed in the MenAfriCar Consortium studies.

#### The prevalence of pharyngeal carriage and the dominant serogroup/genogroup prior to vaccination with MenAfriVac^®^

The prevalence of pharyngeal carriage identified in 40 surveys undertaken prior to the introduction of MenAfriVac^®^, and the dominant *N. meningitidis* serogroup/genogroup, identified in these surveys, are summarised in Table 1. Twenty‐nine of these studies were conducted by members of the MenAfriCar consortium. Both the overall prevalence of carriage and the dominant serogroup/genogroup varied substantially among sites studied at the same time, and at the same site studied at different times. The mean prevalence of pharyngeal meningococcal carriage in 66,707 pharyngeal swabs obtained during 40 carriage surveys was 4.5% [95% CI: 3.4%, 6.8%], varying between surveys from <1% to 24.1%; however, in only six surveys was the meningococcal carriage prevalence 10% or greater (Table 1). In most surveys, capsulated meningococci (mean prevalence 2.8% [95% CI: 1.9%; 5.2%]) were more prevalent than non‐capsulated meningococci (mean prevalence 1.8% [95% CI: 1.0%, 2.4%]) (Table 1) but the proportion of non‐capsulated meningococci varied substantially among sites and over time. The highest prevalence of non‐capsulated meningococci was found in Ethiopia 18.

**Table 1:** Prevalence of meningococcal carriage in 40 surveys carried out in the African meningitis belt from 2006 to 2014 prior to the introduction of MenAfriVacR

Country	Date of study	Season	Urban/rural	Epidemic/non‐epidemic	Sampling method	Age group years	Number of subjects	Prevalence of *Neisseria meningitidis*				Ref (Numbers)
								Overall number (%)	Capsulated number (%)	Non‐capsulated number (%)	Dominant Genogroups	
Burkina Faso	2006	Dry	Rural	Epidemic	Cluster age‐categorised	1–39 years	624	134 (21.5)	131 (21.0)	3 (0.5)	A > Y	Mueller *et al*. (2011) 11
	2006	Dry	Rural	Epidemic	DSS	All	316	36 (11.4)	34 (10.8)	2 (0.6)	All Y	Sie *et al*. (2008) 8
					Random selection							
		Dry	Rural	Epidemic	DSS	All	180	24 (13.3)	23 (12.8)	1 (0.6)	A	Sie *et al*. (2008) 8
					Random selection							
	2008	Dry	Urban	Non‐epidemic	Random selection	1–59 years	538	9 (1.7)	6 (1.1)	3 (0.6)	Y>W>x	Trotter *et al*. (2013) 9
	2009	Dry	Rural + Urban	Non‐epidemic	Multi‐stage cluster	1–29 years	5024	206 (4.1)	172 (3.4)	34 (0.7)	Y>W>A	Kristiansen *et al*. (2011) 12
		Dry (late)	Rural + Urban	Non‐epidemic	Multi‐stage cluster	1–29 years	5121	270 (5.3)	245 (4.8)	25 (0.5)	Y>X>A	Kristiansen *et al*. (2011) 12
		Rainy	Rural + Urban	Non‐epidemic	Multi‐stage cluster	1–29 years	5074	171 (3.4)	140 (2.8)	31 (0.6)	Y>X>W	Kristiansen *et al*. (2011) 12
		Rainy	Rural + Urban	Non‐epidemic	Multi‐stage cluster	1–29 years	5107	162 (3.2)	144 (2.8)	18 (0.4)	Y>X>A	Kristiansen *et al*. (2011) 12
	2010	Rainy	Rural + Urban	Non‐epidemic	Multi‐stage cluster	1–29 years	3428	113 (3.3)	102 (3.0)	11 (0.3)	X>Y>W	Kristiansen *et al*. (2011) 12
Chad	2010	Rainy	Urban	Epidemic	Census	All ages	998	8 (0.8)	6 (0.6)	2 (0.2)	A>W=X	MenAfriCar (2015) 16
			Rural		Random selection	All ages	988	6 (0.6)	6 (0.6)	0	ND>A	MenAfriCar (2015) 16
	2011	Rainy	Urban		Random selection	All ages	1046	13 (1.2)	6 (0.6)	7 (0.7)	A	MenAfriCar (2015) 16
			Rural		Random selection	All ages	4261	55 (1.3)	50 (1.2)	5 (0.1)	A	MenAfriCar (2015) 16
Ethiopia	2010	Rainy	Urban	Non‐epidemic	DHSS	All ages	940	53 (5.6)	18 (1.9)	35 (3.7)	ND>Y=C	MenAfriCar (2015) 16
			Rural	Non‐epidemic	Random selection	All ages	944	67 (7.1)	23 (2.4)	44 (4.7)	Y	MenAfriCar (2015) 16
	2011	Rainy	Urban	Non‐epidemic	Random selection	All ages	1011	54 (5.3)	9 (0.9)	45 (4.5)	Y	MenAfriCar (2015) 16
			Rural	Non‐epidemic	Random selection	All ages	1014	69 (6.8)	22 (2.2)	47 (4.6)	Y	MenAfriCar (2015) 16
	2012	Dry	Urban	Non‐epidemic	Random selection	All ages	1034	44 (4.3)	6 (0.6)	38 (3.7)	ND	MenAfriCar (2015) 16
			Rural	Non‐epidemic	Random selection	All ages	1027	103 (10.0)	8 (0.8)	95 (9.3)	Y	MenAfriCar (2015) 16
	2014	Wet	Rural	Non‐epidemic	DSS	1–29 years	7479	492 (6.6)	116 (1.6)	376 (5.0)	X>W	Barnes *et al*. (2016) 18
					Random selection							
Ghana	2010	Rainy	Urban	Non‐epidemic	DHSS	All ages	557	1 (0.2)	0	1 (0.2)	na	MenAfricar (2015) 16
			Rural	Non‐epidemic	Random selection	All ages	602	4 (0.7)	4 (0.7)	0	W	
	2011	Rainy	Urban	Non‐epidemic	Random selection	All ages	1030	6 (0.6)	5 (0.5)	1 (O.1)	W	MenAfricar (2015) 16
			Rural	Non‐epidemic	Random selection	All ages	1001	64 (6.4)	60 (6.0)	4 (0.4)	W	MenAfricar (2015) 16
	2012	Dry	Urban	Non‐epidemic	Random selection	All ages	1007	68 (6.8)	65 (6.5)	3 (0.3)	W	MenAfricar (2015) 16
			Rural	Non‐epidemic	Random selection	All ages	1012	60 (0.6)	55 (5.4)	5 (0.5)	W	MenAfricar (2015) 16
Mali	2010	Rainy	Urban	Non‐epidemic	DHSS	All ages	400	20 (5.0)	2 (0.5)	18 (4.5)	W and Y	Basta *et al*. (2017) 22
	2010	Rainy	Urban	Non‐epidemic	Census	All ages	2405	16 (0.7)	10 (0.4)	6 (0.3)	Y=B>W	MenAfricar (2015) 16
			Rural	Non‐epidemic	Random selection	All ages	2439	10 (0.4)	8 (0.3)	2 (0.1)	W	MenAfricar (2015) 16
Niger	2010	Rainy	Urban	Non‐epidemic	Census	All ages	2433	194 (7.9)	89 (3.6)	105 (4.3)	W	MenAfricar (2015) 16
			Rural	Non‐epidemic	Random selection	All ages	1802	177 (9.8)	22 (1.2)	155 (8.6)	W	MenAfricar (2015) 16
Nigeria	2010	Rainy	Urban	Non‐epidemic	Census	All ages	781	3 (0.4)	3 (0.4)	0	W	MenAfricar (2015) 16
			Rural	Non‐epidemic	Random selection	All ages	739	0	0	0		MenAfricar (2015) 16
	2011	Rainy	Rural	Non‐epidemic	Random selection	All ages	936	0	0	0		MenAfricar (2015) 16
Senegal	2010	Rainy	Urban	Non‐Epidemic	DHSS	All ages	706	14 (2.0)	9 (1.3)	5 (0.7)	C	MenAfricar (2015) 16
			Rural	All ages	Random selection	All ages	708	31 (4.4)	11 (1.6)	20 (2.8)	Y>C	MenAfricar (2015) 16
	2011	Rainy	Urban	All ages	Random selection	All ages	771	11 (1.4)	6 (0.8)	5 (0.6)	Y	MenAfricar (2015) 16
			Rural	All ages	Random selection	All ages	909	41 (4.5)	17 (1.9)	24 (2.6)	Y>C	MenAfricar (2015) 16
	2012	Dry	Urban	All ages	Random selection	All ages	453	52 (11.5)	50 (11.0)	2 (0.4)	W	MenAfricar (2015) 16
			Rural	All ages	Random selection	All ages	862	208 (24.1)	205 (23.8)	3 (0.3)	W	MenAfricar (2015) 16
							67707	3069 (4.5)	1888 (2.8)	1181 (1.7)		

The highest carriage rate reported (24.1%) among the studies conducted by the MenAfriCar Consortium was from a rural community in Senegal predominantly due to a sudden increase in the prevalence of carriage with serogroup W meningococci (*Nm*W) 25. Serogroup Y meningococci (*NmY*) predominated in one area of Ethiopia 16 and *NmX* and *NmY* in another area of the same country 18. A predominance of serogroup A meningococci (*Nm*A) was detected only in Chad in 2010 and 2011 18 and in Burkina Faso in 2006 11; these three surveys were undertaken at the time of a serogroup A epidemic. The prevalence of carriage of *Nm*A in Chad was only about 1%, even though a major epidemic was occurring 4. In Burkina Faso, significant differences in the carriage prevalence were observed between nearby villages 12 and the same variation in patterns of carriage over short distances was noted in Ethiopia 18.

#### Transmission of *N. meningitidis* within households

A pilot study of household transmission in Mali recruited 20 households and followed them for 6 months prior to MenAfriVac^R^ introduction, followed by a further three, monthly visits 10 months after vaccination 24. The acquisition rate of meningococci was 2.3 per 100 individuals per month and the mean duration of carriage was 2.9 months. A large majority of isolates in this study were non‐capsulated.

In a subsequent larger study conducted in five countries by members of the MenAfriCar consortium, a pharyngeal swab was collected from consenting household members within a month of detection of an index carrier and then at fortnightly intervals for 2 months and then monthly for a further 6 months 19. In 51 households, the initial identification of a meningococcal carrier was not confirmed by molecular characterisation and these households formed a control group. One hundred and fifty‐two (20.6%) of the 739 residents of households with an initial carrier acquired a meningococcus during the 6 months of follow‐up compared with 35/371 (9.4%) of residents of control households (*P* < 0.001). Transmission from sibling to sibling was the most frequent pattern of household transmission. The mean duration of carriage was 3.4 months and only 20 individuals carried meningococci for the full 6‐month period of follow‐up 19.

#### Risk factors for carriage of *N. meningitidis*

Six studies conducted by independent groups of investigators reported on risk factors for the prevalence of pharyngeal carriage of *N*. *meningitidis* and one study investigated risk factors for acquisition (Table 2) 6, 9, 18, 22, 23. Age was a strong risk factor for carriage, with the prevalence of carriage peaking in those aged 5–14 years in nearly all surveys. Carriage was more frequent in males than in females. Other risk factors varied between surveys (Table 2) but included (i) the presence of an internal kitchen or a cigarette smoker in the household; (ii) household crowding; (iii) a recent respiratory infection (iv) living in a rural environment and (v) a past history of vaccination with a polysaccharide meningococcal vaccine. The latter was an unexpected finding, which may have been due in part to the fact that polysaccharide vaccination, which has little or no impact on carriage, is likely to have focussed on those communities most at risk. In four studies, a 50% increase in carriage was detected in the dry season compared to the rainy season and a similar increase was noted in a further unpublished study from Burkina Faso (Kristiansen, personal communication).

**Table 2:** Risk factors for carriage of *Neisseria meningitidis* identified during cross‐sectional pharyngeal carriage surveys conducted in the African meningitis belt which have been reported since 2007

Characteristics of survey									
Year	2003	2006		2009		2009	2010–2011	2010–2012	2017
Country/countries	Burkina Faso	Burkina Faso		Burkina Faso		Burkina Faso	Burkina Faso	Chad, Ethiopia, Ghana, Mali	Ethiopia
								Niger, Nigeria, Senegal	
Epidemic/non‐epidemic	Non‐epidemic	Epidemic		Non‐epidemic		Non‐epidemic	Non‐epidemic	Non‐epidemic except Chad	Non‐epidemic
Season	Dry	Dry		Combined		Combined	Combined	Combined	Dry
Number of participants/swabs	488	617				20 326	25 521	15 512	240
Age group	4–29 years	2–29 years		1 year or >		1–29 years	1–29 years		6–16 years
Study end‐point	Longitudinal survey	Cross ‐sectional survey		Cross‐sectional survey		Cross‐sectional survey	Cross‐sectional survey	Cross‐sectional survey	Cross‐sectional survey
Analysis	Logistic regression	Logistic regression		Logistic regression				Logistic regression	Logistic regression
Serogroup	Non‐groupable	Group A	Group Y	Group X	Group Y	All capsulated and non‐groupable	All capsulated and non‐groupable	All capsulated and non‐groupable	All capsulated and non‐groupable
Number of carriers	81	95	35	213	70	809	1649	1687	49
Risk Factors	Acquisition	Carriage	Carriage	Carriage	Carriage			Carriage	
Age group		Highest 4–29 years		Highest 5–14 years	Highest 5–14 years			Highest 5–14 years	
Male sex		OR 2.41 (1.42–4.09)		OR 1.19 (0.78–1.83)	OR 1.21 (0.98–1.50)	*P* < 0.05	*P* < 0.001	OR = 1.17 (1.10, 1.24)	
Dry season				OR 1.51 (1.06–2.16)	All *Nm* (*n* = 419)	*P* < 0.001	*P* < 0.001	OR = 1.54 (1.37, 1.75)	
High relative humidity	IRR 2.18 (1.28–3.71)								
Student	IRR 0.49 (0.29–0.84)			OR 1.37 (0.34–5.53)	OR 1.26 (0.92–1.73)				
Occupation									
Education									
Rural *vs*. urban						*P* < 0.001		OR = 1.44 (1.28, 1.63)	
House quality									
Crowding		OR 0.44 (0.22–0.88)*	OR 5.85 (1.67–20.44)†					OR = 1.24 (1.09, 1.42)‡	OR = 1.26 (1.18, 1.35)*
Kitchen location inside	IRR 0.63 (0.37–1.06)							OR = 1.35 (1.12, 1.63)	
Exposure to cigarette smoke			OR 2.51 (1–16–5.45)					OR = 1.16 (1.02, 1.33)	
Meningitis in household		OR 2.53 (1.34–4.76)							
Recent respiratory infection		OR 3.41 (1.79–6.48)							
Current rhinitis		OR 2.56 (1.26–5.21)							
Recent medication				OR 1.56 (0.81–3.03)	OR 1.14 (0.78–1.67)				
History of recent meningococcal vaccination				OR 1.96 (1.01–3.78)	OR 1.05 (0.74–1.49)			OR = 0.72 (0.60. 0.85)	
Reference	Mueller *et al*. (2008) 10			Ba *et al*. (2014) 15		Kristiansen *et al*. (2011) 12	Kristiansen *et al*. (2013) 6	MenAfriCar (2015) 16	Alemayehu *et al*. (2017) 23

Boxes highlighted in red indicate an increased risk of meningococcal carriage, those in green a decreased risk. Risks are presented as Incidence rate Ratios (IRR) or Odds Ratios (OR) with 95% confidence intervals.

* >5 sharing bedroom.

† >11 sharing evening meal.

‡ >2 people/room.

Blood samples collected in the household study described above allowed an evaluation of the association between serum bactericidal antibody (SBA) concentration and acquisition of carriage during the following 6 months. Sufficient episodes of transmission of *Nm*W and *NmY* occurred within the study households to allow correlations to be made between SBA titre to *Nm*W and *Nm*Y and acquisition of carriage with meningococci of the respective serogroup. No correlation was found, even though some subjects had high SBA titres in the range of those found following vaccination 28.

#### Impact of MenAfriVac^R^ on the prevalence of carriage

**Figure 2:**
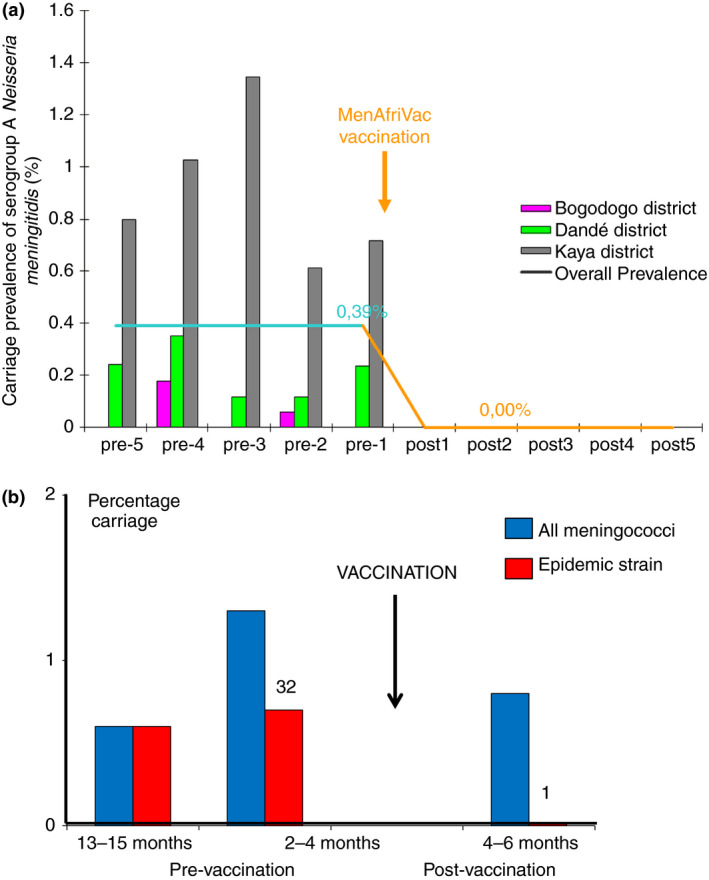
The impact of group A meningococcal conjugate vaccine MenAfriVac^R^ on pharyngeal carriage of the group A meningococcus in (a) Burkina Faso and (b) Chad 4. Figure 2a is modified from reference 5 with permission.

Because of the infrequent detection of *Nm*A during the period of the studies conducted since 2007, it was possible to study the impact of the MenAfriVac^R^ on carriage of *Nm*A in only two countries (Figure 2). In Burkina Faso, the carriage prevalence of *Nm*A was low before the introduction of the vaccine (<1%) but fell to zero a month after vaccination 6; *Nm*A was detected in only one carrier (0.02%) during a survey conducted 2 years later (Figure 2a) 5. In Chad, the prevalence of *Nm*A prior to vaccination was also low (<1%) but 4–6 months after vaccination only one group A meningococcus was found in the 5001 subjects studied (Figure 2b) 4. In Burkina Faso, there was an increase in NmX and NmY carriage and in Chad an increase in the prevalence of carriage with capsule null *N. meningitis* following vaccination with MenAfriVac^R^. A study undertaken in 240 school‐age children resident in an urban area near to Addis Ababa, after national implementation of MenAfriVac^R^, and which employed nasopharyngeal rather than oropharyngeal swabs found a high overall prevalence of meningococcal carriage (20.4%) with a predominance of serogroups W and C 23.

#### Molecular epidemiology of *Neisseria* species in the African meningitis belt

The majority of the carriage isolates of *N. meningitidis* identified during pre‐MenAfriVac^R^ carriage surveys conducted in Burkina Faso were *NmY* (457) 14. Other serogroups identified in Burkina Faso were *Nm*W (70), *Nm*X (90), *Nm*A (80) and *Nm*C 14. One hundred and eight isolates were not serotypable. Twenty‐nine different sequence types, five different clonal complexes and 51 PorA:FetA combinations were identified, allowing the characterisation of 68 distinct strain types [defined as serogroup:PorA VR1,VR2:FetA VR: ST(cc)]. The *Nm*A isolates were all A:P1.20,9:F3‐1:ST‐2859(cc5), the *NmW* were all from cc175, the *NmC* from cc41/44, the *NmY* belonged to two different clonal complexes (cc23 and cc167) while the *NmX* belonged to ST‐181, previously unassigned to any clonal complex. Although multiple *porA‐fetA* combinations were identified for all but serogroup A, one combination was found in the majority of isolates for each serogroup suggesting selection pressure for particular genetic traits. The surveys conducted in the same sites a year after MenAfriVac^R^ vaccination identified mostly *NmX* belonging to ST‐181 (5.33% of the samples) 14. Two years after MenAfriVac^R^ vaccination, *Nm*W from ST‐11 cc dominated with a carriage prevalence of 6.85% 5.

In Ethiopia, a carriage study undertaken prior to the introduction of MenAfriVac^R^ found a predominance of non‐capsulated meningococci identified as ST‐192; the two other serogroups identified most frequently were *NmX* from cc181 and cc41/44, and *NmW* from the hypervirulent cc11 clonal complexes. Low carriage prevalences of *NmC*:cc103 and *NmY*:cc167 meningococci were also identified. No *Nm*A was detected. The diversity of the isolates remained low but higher than that recorded in the Burkina Faso studies 18.

The characterisation method used in the MenAfriCar studies did not include MLST; instead, sequencing of a fragment of the *rplF* ribosomal locus was conducted to identify the meningococcus, followed by PorA VR1 and VR2 finetyping and genogrouping by PCR and *cnl* sequencing 25. The most frequently identified meningococci were non‐capsulated followed by *Nm*W with a prevalence of 1.3%. A total of 132 strain types, defined as the combination of the genogroup and porA subtype, were identified; the most frequently identified were cnl:P1.18‐11,42‐11, usually associated with ST‐192, and *NmW*:P1.5,2, usually associated with the hypervirulent cc11 16.

Post MenAfriVac^R^ vaccination changes included a marked increase in the overall prevalence of the *cnl:* P1.18‐11,42‐1 strain in Chad 4 and an expansion of the *Nm* W:P1.5,2 strain in Mali and Niger 16.

Few studies of whole genome sequencing of meningococcal carriage isolates obtained in the African meningitis belt have been reported. A study of *Nm*A isolates, including both carriage and invasive isolates, collected during an epidemic in Chad demonstrated differences between closely related strains that were indistinguishable by MLST analysis 21. The bacterial clusters identified were linked to age group but did not differ between carriage and invasive disease isolates. A study from Ethiopia compared the genomes of paired isolates collected at an interval of 2 months within the same 50 individuals and showed changes in genes belonging to the pilin family, the restriction/modification systems, the opacity proteins and genes involved in glycosylation 22.

#### Carriage of non‐meningococcal Neisseria species

Three studies investigated carriage with non‐meningococcal *Neisseria* species, two focussed only on *N. lactamica* whilst the third studied the prevalence of additional *Neisseria* species.

In Burkina Faso, the prevalence of *N. lactamica* in 1–29 year olds in 45 847 samples obtained during nine carriage surveys conducted from 2009 to 2011 was remarkably constant at about 20% and differed little between three study sites 13. The carriage rate peaked at a prevalence of 40.1% in 2 year olds and then fell progressively to about 10% in 10–29 year olds. Carriage of *N. lactamica* was slightly more prevalent in males than in females (OR = 1.11 [95% CI: 1.04–1.18]) but the carriage rate did not vary by season or change following the introduction of MenAfriVac^R^. MLST was applied to a selection of *N. lactamica* isolates; however, high genetic diversity was observed with 62 different genotypes identified 13.

Carriage of *N. lactamica* was also studied in a rural community in Ethiopia in 2014 18. The prevalence of carriage in subjects aged 1–29 years was similar to that seen in Burkina Faso (28.1%) but, unlike the situation in Burkina Faso, varied significantly among districts (kebeles). The highest prevalence of carriage (54.5%) was seen in 1‐year‐old children. No difference was found between genders and there was no evidence of an interaction between *N. lactamica* and *N. meningitidis*.

During the course of the MenAfriCar surveys conducted in seven meningitis belt countries, non‐meningococcal *Neisseria* were detected in 3,015 of 46 034 (6.6%) pharyngeal swabs 17. *Neisseria lactamica* was the most frequently isolated non‐meningococcal *Neisseria* species, with an overall prevalence of 5.6% (95% CI 5.3, 5.8) in all age groups and a peak prevalence of 14.0% in those aged 1–4 years. Prevalence varied significantly among countries from 1.9% (95% CI: 1.6, 2.2) in Mali to 13.3% (95% CI: 12.5, 14.10) in Niger. Small numbers of other non‐meningococcal *Neisseria* were isolated – *Neisseria polysaccharea* (290), *Neisseria bergeri* (113) and *Neisseria subflava* (24). *Neisseria polysaccharea* was found most frequently in Ethiopia whilst *N. bergeri* and *N. subflava* were isolated most frequently in Chad. Significant risk factors for carriage of any non‐meningococcal *Neisseria* species included age, gender and season 17. Carriage was less prevalent in males than in females (RRR = 0.87 [0.80, 0.94]) and less prevalent in the dry season than in the rainy season (RRR = 0.78 [0.70, 0.86]), in contrast to carriage of *N*. *meningitidis* which was more prevalent in the dry season (RRR = 1.53 [1.35, 1.74]). Molecular characterisation of the non‐meningococcal *Neisseria* species using a combination of Sanger sequencing of a fragment of the *rplF* locus and of the variable region of the *fetA* locus identified five different *Neisseria* species and 636 combinations of *rplF‐*FetA VR, highlighting the large diversity of the *Neisseria* species identified in the region 17.

## Discussion

This review has brought together the results of meningococcal carriage surveys conducted in the African meningitis reported since 2007. In comparison with earlier studies, whose methods were very varied, these surveys used very similar field and laboratory methods allowing more reliable comparisons of changes in the pattern of meningococcal carriage in time and place than was the case in the period prior to the 2007 review.

The combined results of a large number of surveys are largely in agreement with the main findings of the earlier studies, in particular the marked differences in the prevalence and serogroup distribution of meningococcal carriage between adjacent sites and over time at the same site. As noted in previous studies, carriage was seen most frequently in those aged 5–14 years, in males more frequently than in females and in those exposed to smoke or a respiratory infection. A new epidemiological finding, not identified clearly in previous studies, was a modest increase in the prevalence of carriage in the dry season, noted in four studies, suggesting an increase in transmission at this time of the year. This could contribute to the seasonal nature of epidemics, as suggested by a mathematical model 29 although it is likely that other microbiological, environment and social factors also play a significant role in the generation of epidemics.

The prevalence of meningococcal carriage varied markedly between surveys but the mean carriage prevalence of 4.4% obtained across the surveys conducted prior to the deployment of MenAfriVac^®^ was lower than that found in most surveys conducted in industrialised countries 30 suggesting that the force of infection is less in the African meningitis belt than in industrialised countries resulting in less naturally acquired immunity and a predisposition to epidemics. This hypothesis is supported by the fact that the diversity of carriage isolates was less in countries of the meningitis belt, especially those in the centre of the meningitis belt, than usually found in industrialised countries. However, the mean duration of carriage detected in a household study was approximately 3 months, a similar duration to that obtained in an earlier study in Nigeria 31, substantially less than that usually found in carriage studies conducted in industrialised countries 32. This provides a potential explanation of why the prevalence of meningococcal carriage is lower in meningitis belt than in industrialised countries.

As reported previously, the prevalence of group A meningococcal carriage was reduced substantially following the introduction of MenAfriVac^®^ in Burkina Faso and Chad, and the impact on this vaccine on carriage is likely to have contributed to profound reductions in group A disease observed across the belt since MenAfriVac^®^ roll‐out 33.

Little was known previously about the pattern of transmission of *N. meningitidis* in African meningitis belt countries. The MenAfriCar study undertaken in five countries found strong evidence for transmission within and outside households 19. This provided important new information of relevance to potential control interventions. For example, it supports the results from a recently conducted village randomised trial of chemoprophylaxis which showed that village‐wide but not household‐targeted prophylaxis was effective in reducing the meningitis attack rate 34.

New molecular methods of detecting and characterising *Neisseria* species have become available since the time of the 2007 review 7. Nevertheless, all the studies conducted since that time have used conventional microbiological methods for the primary identification of *N. meningitidis* but the majority have then used molecular methods to characterise these further including determining capsular group. Several studies reported a lack of congruence between typing by the conventional agglutination method and PCR and the former method, which requires skill and experience, is likely to become only second choice for grouping as access to PCR become more widely available. The size and cost of such surveys could be reduced if more sensitive methods of detecting meningococci in the pharynx could be developed. Thus, the results of a pilot study conducted in The Gambia which used preliminary culture of a swab in broth followed by PCR as a supplement to the conventional microbiological approach is important as this showed the broth culture method was substantially more sensitive than conventional culture 20. Similar approaches, including direct isolation of DNA from swabs, are likely to be used increasingly frequently in surveys conducted in the meningitis belt. Molecular methods also have the advantage of allowing quantitation of the density of carriage, a factor that could be important in determining the likelihood of transmission.

Because the carriage prevalence of *N. meningitidis* is generally low, and conventional isolation techniques relatively insensitive, large studies are needed to measure it precisely and to make comparisons among groups, for example, those in vaccine trials. The introduction of new, potentially more sensitive diagnostic methods may help to reduce required sample sizes, but conducting high quality carriage surveys will still be demanding, especially in settings where technical and financial resources are constrained. For these reasons it is important to demonstrate the potential value of carriage studies, especially in areas with few resources, such as the African meningitis belt. An important role for carriage studies, especially when performed together with molecular characterisation of meningococcal isolates, is identification of patterns of transmission of *N. meningitidis*. Remarkably, few such studies have been undertaken to date and yet this information would be of very valuable in the rational planning of vaccination and/or chemoprophylaxis interventions. A second key role for carriage studies is determining the impact on carriage of novel vaccines, such as the pentavalent meningococcal conjugate vaccine currently under evaluation. It is now clear that the ability to prevent pharyngeal acquisition of meningococci is a key attribute of conjugate vaccines and that vaccines that are unable to do this will be less effective than those that can. Finally, comparison of representative carriage and disease isolates that have been characterised in detail with molecular methods, including WGS, provide important information on the epidemiology, genetics and pathogenesis of the meningococcus. These data will be essential in understanding how hyperinvasive meningococci evolve.

The majority of carriage studies have focussed on *N meningitidis* alone (with a minority also including *N lactamica*). More recent studies have shown that this bacterium is only one of a number of *Neisseria* species resident in the pharynx of inhabitants of the meningitis belt. The prevalence of these bacteria also varies by time and space.

## Supporting information


**Table S1.** Details of the 20 papers identified as providing information on meningococcal carriage surveys in the African meningitis belt since 2007.
